# ﻿*Paraboeazunyiensis* (Gesneriaceae), a new species from north Guizhou, China

**DOI:** 10.3897/phytokeys.235.111412

**Published:** 2023-11-06

**Authors:** Tan Deng, Fang Wen, Da-Jun Xie, Ruo-Xun Wei, Lin He, Quan-Li Dou, Zheng-Min Qian, Ren-Bo Zhang

**Affiliations:** 1 Department of Biology, Zunyi Normal College, Zunyi, CN-563000 Guizhou, China Zunyi Normal College Zunyi China; 2 Guangxi Key Laboratory of Plant Conservation and Restoration Ecology in Karst Terrain, Guangxi Institute of Botany, Guangxi Zhuang Autonomous Region and Chinese Academy of Sciences, CN-541006 Guilin, Guangxi, China Guangxi Institute of Botany, Guangxi Zhuang Autonomous Region and Chinese Academy of Sciences Guilin China; 3 Gesneriad Committee of China Wild Plant Conservation Association (GC), National Gesneriaceae Germplasm Resources Bank of GXIB (NGGRB), Gesneriad Conservation Center of China (GCCC), CN-541006 Guilin, Guangxi, China National Gesneriaceae Germplasm Resources Bank of GXIB Guilin China; 4 Sichuan Academy of Forestry, CN-610081, Chengdu, China Sichuan Academy of Forestry Chengdu China

**Keywords:** Flora of Guizhou, lithophyte, new taxon, *Paraboeacrassifolia*, taxonomy

## Abstract

A new lithophytic species, *Paraboeazunyiensis* T.Deng, F.Wen & R.B.Zhang (Gesneriaceae), inhabiting Karst rocks in northern Guizhou, China, is introduced and depicted in this study. It bears a resemblance to *P.crassifolia* (Hemsl.) B.L. Burtt, yet is distinguishable by its shorter filaments and staminodes, triangular ovate calyx segments, and ovaries surpassing the styles in length. Moreover, the phylogenetic tree constructed from nuclear DNA (ITS) and plastid DNA (*trnL-F*) data firmly support the differentiation of this novel species from *P.crassifolia*.

## ﻿Introduction

*Paraboea* (C.B.Clarke) Ridl. was first published by Clarke as a section, Didymocarpussect.ParaboeaClarke (1883) (Xu et al. 2008), and was elevated to generic level by Ridley (1905). Burtt (1984) defined *Boea* and *Paraboea* based on differences in indumentum (simple straight hairs in *Boea* and interwoven arachnoid-like hairs in *Paraboea*). *Paraboea* comprises ca. 130 species characterized by abaxially matted leaves with densely interwoven indumentum and flowers featuring flat-faced to shortly campanulate corolla and non-erect anthers (Guo 2016; Xu et al. 2017). At present, this genus is a member of the tribe Didymoearpeae, subfamily Cyrtandroideae, family Gesneriaceae (Wang et al. 1990), and is primarily distributed in Karst habitats in China. Exceptions include *Paraboeacrassifila* W.B.Xu & J.Guo (exclusively found in the Danxia landscape of Rong County, Guangxi, China) (Guo 2016) and *P.sinensis* (Oliv.) B.L.Burtt (found in both the Karst and Danxia landscapes) (Wei 2018; Wei et al. 2022).

Firstly, in China, *Paraboea* was initially recognized with 18 species, most of which exhibit narrow endemism (Wang et al. 1998; Fu et al. 2004). The southern region of China hosts a rich diversity of *Paraboea* species. Over ten new species have been reported since 2004 across various provinces: Guangxi (Xu and Wei 2004; Chen et al. 2008; Xu et al. 2012; Wen and Wei 2016), Guangdong (Wen et al. 2013; Wen and Wei 2016), Yunnan (Chen et al. 2012; He et al. 2018; Zhang et al. 2020), Hunan (Wen and Wei 2016), and Guizhou Province (Wen and Wei 2016; Guo et al. 2020).

In April 2023, a *Paraboea*-like species that was morphologically similar to *P.crassifolia* (Hemsl.) B.L.Burtt. was discovered in Guizhou Province, China. Through meticulous analysis of flowering specimens in the laboratory and detailed observation of live plants to compare vegetative and reproductive organs, significant distinctions between the two species became evident. The application of ITS and *trnL-F* for phylogenetic analysis further validated the distinctness of the new species from *P.crassifolia*. As a result, a conclusion was reached, designating it as a novel species within the realm of scientific understanding.

## ﻿Materials and methods

### ﻿Taxonomic revision

The studied specimens were obtained from the type locality and deposited in the Botany Herbarium at Zunyi Normal College (ZY) and the Guangxi Institute of Botany Herbarium (IBK). Using a stereomicroscope (Olympus Optical Microscope SZ61, Olympus Corporation, Japan), we conducted micromorphological analyses and photography. We compared the morphological traits with the protologue and type specimens of previously described *Paraboea* species, especially new *Paraboea* taxa from Guizhou and nearby provinces, along with herbarium specimens at relevant herbaria (e.g., IBK, IBSC, KUN, PE, and ZY).

### ﻿Phylogenetic analysis

Leaf material of the undescribed species was collected in Maoli Town, Zunyi City (Guizhou, China) and promptly silica-dried for DNA extraction. The nuclear ribosomal internal transcribed spacer (ITS) region and plastid *trnL-F* intron spacer region (trnL-F) were utilized in the study. Following Weber et al. (2011), we employed primers, conducted DNA extraction, PCR amplification and sequencing. To elucidate the genus’s phylogenetic affinities, we integrated 36 *Paraboea* species (Table 1). Three former *Boea* species, *Damrongiaclarkeana* (Hemsl.) C.Puglisi, *Dorcocerashygrometrica* Bunge and *Dorcocerasphilippinense* Schltr., were selected as outgroups based on prior phylogenetic analyses (Möller et al. 2011; Guo 2016).

**Table 1.** The GenBank accession numbers used in this study.

Species name	ITS	*trnL-F*
*Damrongiaclarkeana*	KJ475430	KM232645
*Dorcocerashygrometrica*	FJ501319	FJ501476
*Dorcocerasphilippensis*	HQ632953	HQ632862
*Paraboeaacutifolia*	JN934753	FJ501464
*Paraboeaamplifolia*	JN934754	JN934712
*Paraboeaburttii*	JN934756	JN934714
*Paraboeacapitata*	FJ501315	AJ492298
*Paraboeaclarkei*	JN934757	JN934715
*Paraboeacrassifolia*	KU203970	FJ501472
*Paraboeadictyoneura*	KJ475415	FJ501463
*Paraboeadivaricata*	JN934759	JN934717
*Paraboeaeffusa*	JN934760	JN934718
*Paraboeaglabra*	JN934761	JN934719
*Paraboeaglabrescens*	JN934785	JN934743
*Paraboeaglabrisepala*	JN934762	JN934720
*Paraboeaglanduliflora*	JN934763	JN934721
*Paraboeaglandulosa*	JN934784	JN934742
*Paraboeaglutinosa*	JN934764	JN934722
*Paraboeahainanensis*	MF315101	MF315107
Paraboeaharrovianavar.ovata	JN934765	JN934723
*Paraboeahavilandii*	JN934766	JN934724
*Paraboeaincudicarpa*	JN934767	JN934725
*Paraboeamartinii*	MF358702	MF358718
*Paraboeaneurophylla*	JN934769	JN934727
*Paraboeapaniculata*	JN934770	JN934728
*Paraboeaparamartinii*	JN934771	JN934729
*Paraboearufescens*	JN934772	FJ501469
*Paraboeasinensis*	JN934773	FJ501474
*Paraboeasubplana*	JN934786	JN934744
*Paraboeasuffruticosa*	JN934774	JN934732
*Paraboeaswinhoei*	JN934775	JN934733
*Paraboeatarutaoensis*	JN934776	JN934734
*Paraboeatrachyphylla*	JN934777	JN934735
*Paraboeatrisepala*	JN934778	JN934736
*Paraboeaumbellata*	JN934779	JN934737
*Paraboeavelutina*	JN934780	MF358725
*Paraboeaverticillata*	JN934781	JN934739
*Paraboeavulpina*	JN934782	JN934740
***Paraboeazunyiensis* sp. nov.**	OR125066	OR123588

Bayesian inference was implemented using MrBayes v3.2.6. Prior to the Bayesian analysis, the mrModelTest v1.0 incorporating the Akaike information criterion (AIC) was used for selecting the best-fit molecular evolution model (GTR+I+G for the ITS and GTR for the *trnL-F*). Homogeneity testing was conducted via PAUP4 software (https://paup.phylosolutions.com/) yielding a *p* value < 0.05, thus prompting the merger of the two regions for subsequent analyses. The BI analyses entailed four Markov Chain Monte Carlo (MCMC) chains, with tree sampling every 100 generations for 2,000,000 generations from a random tree. Upon stabilizing log-likelihood scores, a consensus tree was computed, excluding 5,000 sampled trees as burn-in (Xie et al. 2014). Tree visualization was carried out in FigTree v.1.4.3 (http://tree.bio.ed.ac.uk/software/figtree/).

## ﻿Taxonomic treatment

### ﻿ Paraboea zunyiensis

Taxon classificationPlantaeLamialesGesneriaceae

T.Deng, F.Wen & R.B.Zhang sp. nov.

E1932C50-732F-560C-B1A0-BF7FBC13C093

urn:lsid:ipni.org:names:77330011-1

Figs 1 , 2

#### Diagnosis.

*Paraboeazunyiensis* morphologically resembles *P.crassifolia*, but can be distinguished by the shorter staminodes (< 1 mm *vs.* 2–2.5 mm in *P.crassifolia*, following the same order), filaments (ca. 1 mm *vs.* (3–) 5.5–7 mm) and anthers (1.5–2.3 mm *vs.* 2.5–3 mm), calyx lobe shape (triangular ovate *vs.* narrowly triangular to linear), the outer calyx indumentum (tomentose *vs.* puberulent or velutinous), and the ovary length (ovaries longer than the styles *vs.* ovaries shorter than the styles).

**Figure 1.**
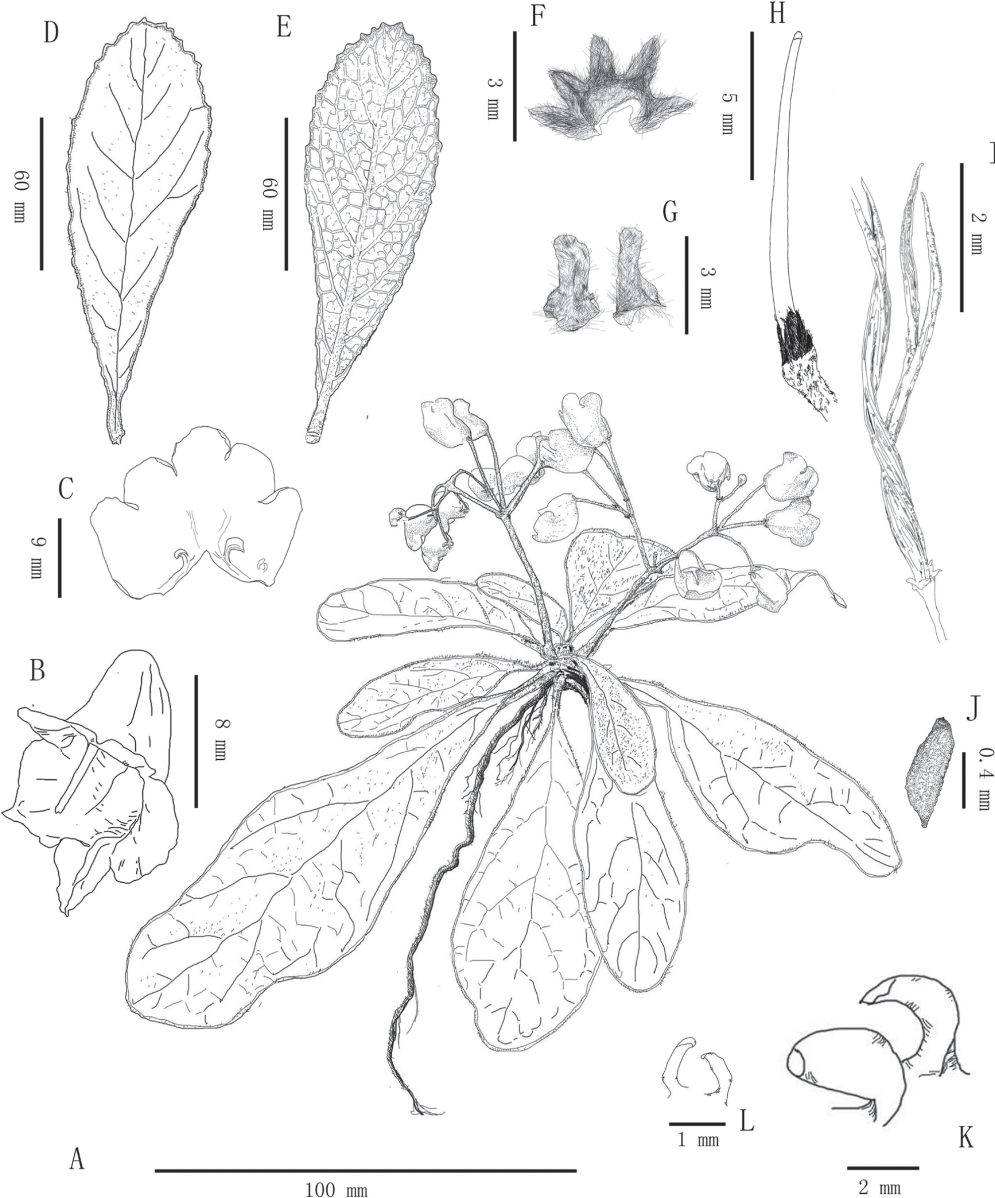
Line drawing of *Paraboeazunyiensis* T.Deng, F.Wen & R.B.Zhang, sp. nov. **A** flowering plant **B** corolla **C** opened corolla **D** adaxial leaf surface **E** abaxial leaf surface **F** calyx **G** bracts **H** pistil **I** capsules **J** seed **K** fertile stamens **L** staminodes. Drawings by Tan Deng from the type specimens.

#### Type.

China, Guizhou Province, Zunyi City, Maoli Town, Xiazhai Valley, elev. ca. 1000 m, 27.36986425°N, 107.05679454°E, growing on the Karst rocks alongside the stream. 15 April 2023, *Ren-Bo Zhang ZRB2493* (fl.) (***holotype***: IBK!, ***isotypes***: ZY!) and 27 May 2023 *Ren-Bo Zhang ZRB2498* (fr.) (***paratype***: ZY!).

**Figure 2.**
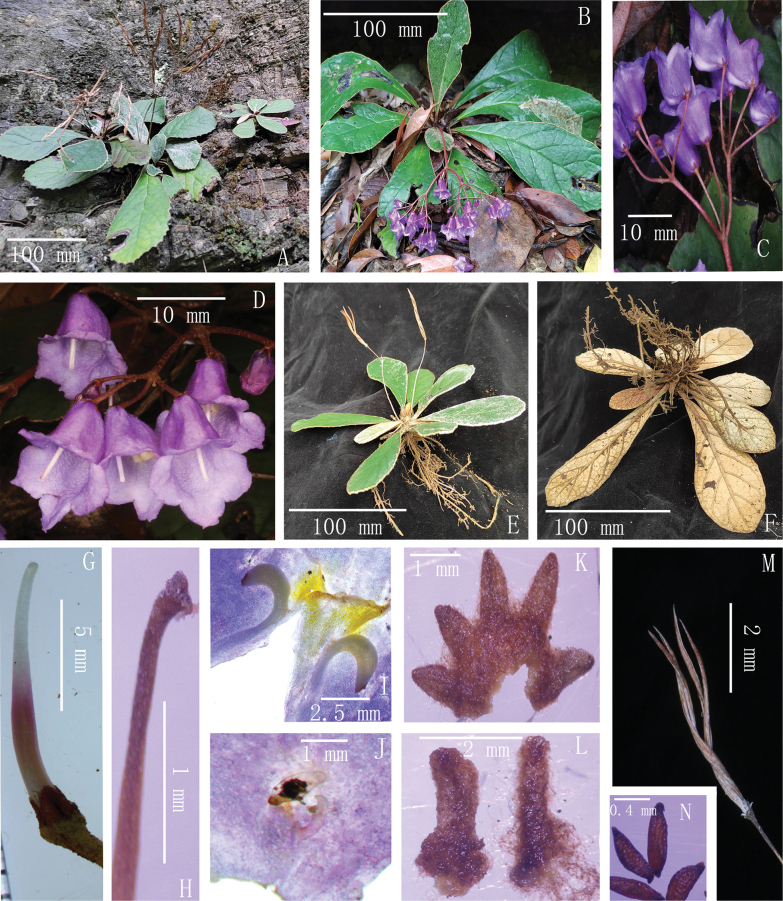
Living or re-watered photographs of *Paraboeazunyiensis* T.Deng, F.Wen & R.B.Zhang, sp. nov. **A** habitat **B** flowering plant **C** cyme **D** flowers **E** fruiting plant **F** upward view of the plant **G** pistil **H** stigma and style **I** fertile stamens **J** staminodes **K** calyx **L** bracts **M** capsules **N** seeds (Photographed by T. Deng and R.B. Zhang)

#### Description.

***Herbs***, stemless. ***Leaves*** basal, petiole 0.8–1.5 cm long; leaf blade spathulate or oboval-oblong, 3.5–12 (–25) × 1–3.5 (–8) cm, thick papery to leathery, adaxially cobwebby-woolly, glabrescent, abaxially densely cobwebby-woolly to pannose, base attenuate to cuneate, margin crenate or subentire, involute, apex rounded or acute; lateral veins 4–7 on each side of midrib. ***Cymes*** 1–2, axillary, each cyme 3–7 branched and 9–22 flowered; peduncle 2–6 cm long, ca. 1.5 mm in diam, cobwebby-woolly, glabrescent from upper part; bracts 2, opposite, linear, 2–3 × 0.6–0.9 mm, outside cobwebby-woolly. ***Pedicel*** 1–2 cm long, cobwebby-woolly. ***Calyx*** ca. 3 mm long, 5-sect from near base; segments triangular ovate, 1–2 × ca. 0.5 mm, outside brown tomentose. ***Corolla*** blue-purplish, ca. 1.5 cm long, subglabrous on both sides; tube 7–8 mm long; adaxial lip ca. 3 mm long, lobes 2–3 × ca. 5 mm; abaxial lip ca. 7 mm long, lobes 3–4 × 5–6 mm. ***Stamens*** 2, filaments ca. 1 mm long, glabrous; anthers 1.5–2.5 mm long; staminodes 2, ca. 0.8 mm long. ***Pistil*** glabrous; ovary 4–6 mm long; style 3–5 mm long; stigma capitate. ***Capsule*** spirally twisted, 2–4 cm long, glabrous. ***Seeds*** 0.5–0.7 × 0.2–0.3 mm, reticulate, apiculate or cuspidate at both ends. **Fl**. Apr–May. **Fr**. May–Jun.

#### Phenology.

Flowering occurs from April to May, and fruiting occurs from May to June.

#### Etymology.

The specific epithet is derived from the type locality, Zunyi City, Guizhou Province, China.

#### Vernacular name.

The Chinese name proposed here is “遵义蛛毛苣苔”. Phonetically, it is “Zūn yì zhū máo jù tái”.

#### Distribution and ecology.

The new species is endemic to Guizhou Province and is known only from the type locality, Xiazhai Valley in Zunyi City. It grows on the steep Karst cliff in a valley, at an altitude ca. 1000 m.

#### Conservation status.

*Paraboeazunyiensis* is known only from the type locality, with the individuals estimated to be over thousands of plants. Considering the narrow distribution area, we proposed it as “NT” (near threatened) according to the guidelines for using the IUCN Red List Categories and Criteria (IUCN Standards and Petitions Committee 2022).

#### Taxonomic and phylogenetic notes.

**Figure 3.**
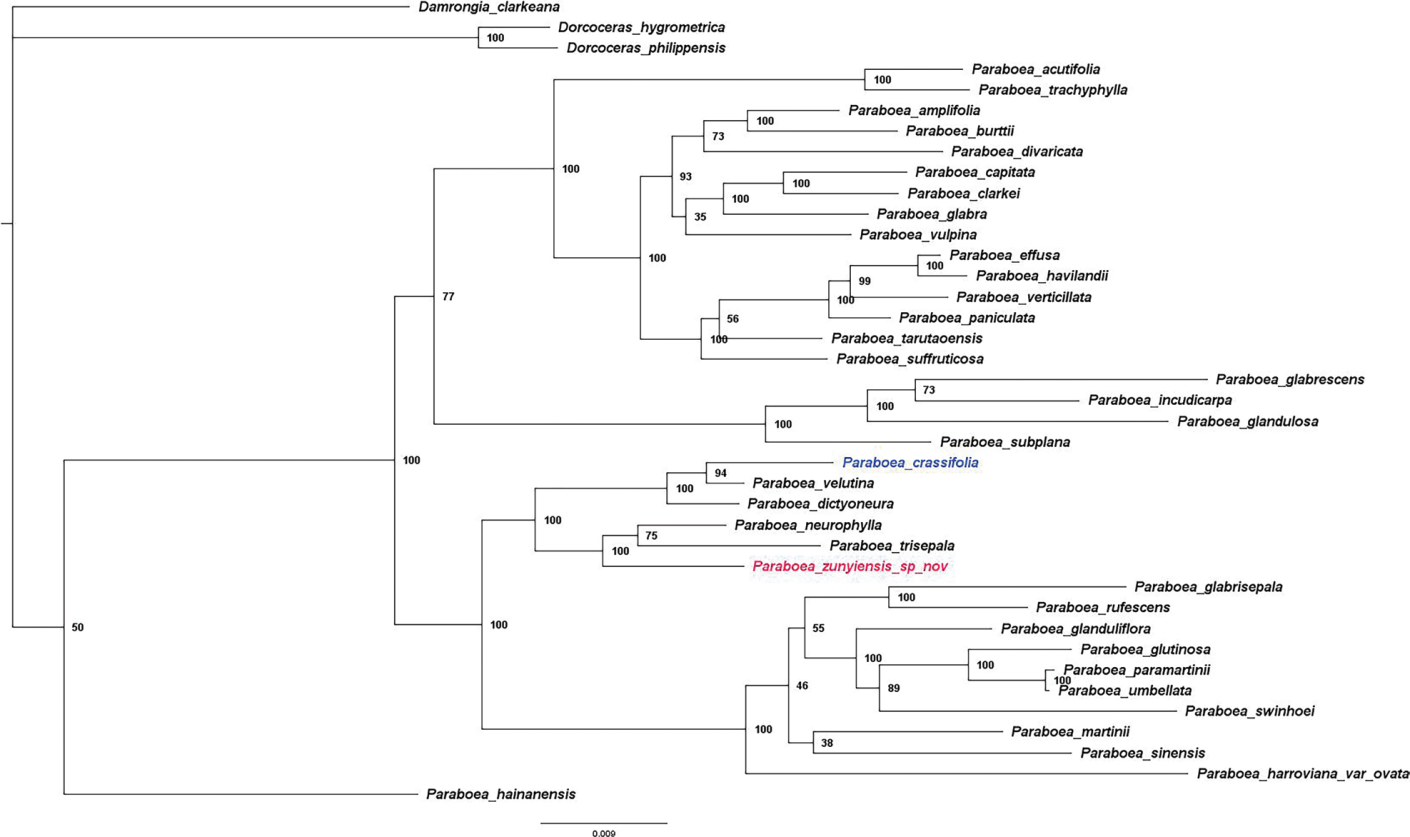
Bayesian phylogenetic tree of *Paraboea* including *P.zunyiensis* based on the combined data from ITS and *trnL-F*, with the branch lengths indicating the nucleotide substitution rates and the posterior probabilities are shown beside the branches.

The aligned matrix of ITS and *trnL-F* sequences comprised 1562 characters. The three outgroup species are clearly distinguishable from the *Paraboea* species (Fig. 3). *P.hainanensis* (Chun) B.L.Burtt is quite different and other *Paraboea* species form two big clades, which matches a previous study (Guo 2016). Although *P.zunyiensis* and *P.crassifolia* are in the same branch (BI = 100%), they are not clustered together and they can be morphologically distinguished from the traits presented in Table 2. *P.zunyiensis* is clustered with *P.neurophylla* (Hance) B.L.Burtt and *P.trisepala* W.H.Chen & Y.M.Shui (BI = 100%). *P.crassifolia* and *P.velutina* (W.T.Wang & C.Z.Gao) B.L.Burtt are clustered as a sister group (BI = 95%) and then clustered with *P.dictyoneura* (Hance) B.L.Burtt.

**Table 2.** Morphological and phenological comparison of *Paraboeazunyiensis* and *P.crassifolia*.

Characters	*Paraboeazunyiensis*	*Paraboeacrassifolia*
Bract width (mm)	0.6–0.9	ca. 0.5
Calyx lobes	triangular ovate	narrowly triangular to linear
Calyx outer indumentum	tomentose	puberulent or velutinous
Filament length (mm)	ca. 1	(3–) 5.5–7
Anther length (mm)	1.5–2.3	2.5–3
Staminode length (mm)	< 1	2–2.5
Ovary (mm)	4–6 (longer than styles)	3–4 (shorter than styles)
Style (mm)	3–5	5.5–6
Fl.	Apr–May	Mar–Jul
Fr.	May–Jun	Sep

## Supplementary Material

XML Treatment for Paraboea zunyiensis

## ﻿Additional information

### Author ORCIDs

Tan Deng https://orcid.org/0000-0002-4575-3298

Fang Wen https://orcid.org/0000-0002-3889-8835

Da-Jun Xie https://orcid.org/0009-0008-4030-4718

Ruo-Xun Wei https://orcid.org/0009-0000-0510-7470

Lin He https://orcid.org/0009-0000-9952-7475

Quan-Li Dou https://orcid.org/0009-0003-8462-7774

Zheng-Min Qian https://orcid.org/0009-0006-7721-966X

Ren-Bo Zhang https://orcid.org/0000-0002-9860-5803

### Data availability

All of the data that support the findings of this study are available in the main text.
